# Prevalence and trends in obesity among Austrian conscripts from 1983 to 2017

**DOI:** 10.1007/s00508-021-01941-9

**Published:** 2021-09-17

**Authors:** Lin Yang, Alfred Juan, Thomas Waldhoer

**Affiliations:** 1 Department of Cancer Epidemiology and Prevention Research, Alberta Health Services, Holy Cross Centre, 2210-2nd Street SW, ACB, T2S 3C3 Calgary, AB Canada; 2 Preventive Oncology & Community Health Sciences, Cumming School of Medicine, University of Calgary, Hospital Drive NW, Calgary, Alberta Canada; 3 Department of Epidemiology, Center for Public Health, Medical University of Vienna, Kinderspitalgasse 15, Vienna, Austria; 4 Ministry of Defence, Vienna, Austria

**Keywords:** Population based, Time trend, Education, Smoking, Body mass index

## Abstract

This article describes the recent prevalence and trend in weight status in young men over three and half decades among Austrian conscripts overall and by subgroups defined by education and smoking behavior. We extracted medical record data from six medical examination stations across the country of all Austrian military conscripts (aged 17–19 years) recruited between 1983 and 2017 (*n* = 1.5 million). Weight and height were measured to calculate body mass index (BMI). Mean BMI increased from 22.7 to 24.3 kg/m^2^ between 1983 and 2017. Over time, the prevalence of obesity (BMI ≥ 30 kg/m^2^) increased from 1.6% (95% CI 1.6–1.7%) to 8.2% (95% CI 8.1–8.3%). The prevalence of obesity among Austrian young men increased remarkably in the past 35 years. Higher levels of education appeared to be associated with lower prevalence of obesity, particularly among the non-smokers.

## Introduction

The contemporary global disease burden is dominated by chronic diseases [1]. The management of chronic diseases is complex given that multiple risk factors may contribute to the development of these diseases. Nevertheless, a number of highly prevalent chronic diseases have excess adiposity as an established cause: type 2 diabetes mellitus, [2, 3] coronary heart disease, [4, 5] high blood pressure, [6, 7] high blood cholesterol, [8] and some cancers [9]. In particular, excess obesity during adolescence and young adulthood increases the risk of obesity [10] and risk of chronic diseases later in adulthood [11, 12].

The most common measure of weight status used to monitor population-wide obesity is the body mass index (BMI), based on which global obesity has nearly tripled since 1975 [13]. The prevalence of overweight and obesity is steadily increasing worldwide, with strong socioeconomic inequalities observed in many developed countries [13].

A number of previous studies reported the weight status in Austria, [14–20] limited to the use of self-reported data, conducted at least a decade ago, and none considered smoking, an important factor influencing obesity. The present study aims to quantify the time trend in weight status in young men over three and half decades using the health examination data from Austrian conscripts. Secondary aim was to explore if the time trend in weight status differs by education and smoking behavior to inform medical practice and public health policy.

## Methods

### Study population

All Austrian men who reach the age of 18 years in a given year are enlisted in that year for either compulsory military or alternative service. The enlistment is preceded by an obligatory medical examination to verify military fitness. Details of this medical examination have been provided elsewhere [21–25]. In brief, the data set contains anonymized data of all young men in Austria in the year they reached age 18 years, including all years from 1983 through 2017. Therefore, this data set represents the total male population. Conscripts between the age of 17 and 19 years at the time of medical investigation were included for the present analyses (8% excluded). We included young men who have complete data on education, weight and height and provinces of residence from the 1983 conscript cohort to the 2017 conscript cohort (9.6% missing). The study design and conduct of the research obtained approval from the Medical University of Vienna Ethics Review Board (ECS 1393/2018).

### Weight status

A total number of six stations in Austria facilitate conscription medical examination, covering 9 provinces: Burgenland + Vienna, Carinthia + Salzburg, Tyrol + Vorarlberg, Styria, Upper Austria and Lower Austria. These routinely collected data are stored in the electronic system of the medical examination stations and are centralized at the Austrian Ministry of Defense.

Height and weight were measured following standard procedures by trained technicians using standardized equipment, with Austrian conscripts wearing underwear and no shoes. Height was measured using a standard anthropometer, and body weight was determined to the nearest 100 g on calibrated scales [21, 24, 26]. The BMI was calculated as weight in kg/(height in meters)^2^. The age range of the Austrian conscripts is around the upper limit of the US Centers for Disease Control and Prevention growth chart, which provides each BMI with a corresponding BMI-for-age percentile (2–19 years) [27]. This approach uses the 5th, 85th and 95th percentiles to define weight category: below 5th percentile (underweight); 5th–< 85th percentiles (normal weight); 85th–< 95th percentile (overweight) and ≥ 95th (obese). In our study sample, the corresponding BMI for these percentiles were 18.3, 26.0, and 30.0 kg/m^2^, which approximate to the standard BMI categories cut-off in adults (≥ 20 years). Therefore, we categorized study participants into standard BMI categories defined by the world health organization: underweight (< 18.5 kg/m^2^), normal weight (18.5–< 25 kg/m^2^), overweight (25–< 30 kg/m^2^), class I obesity (≥ 30.0–<35 kg/m^2^), class II obesity (≥ 35.0–<40 kg/m^2^), and class III obesity (≥ 40 kg/m^2^) [28].

### Individual and social factors

We retrieved data of individual characteristics including smoking and education. Smoking was self-reported with yes/no responses to the question “do you currently smoke?” and classified into smokers (yes) and non-smokers (no). Data on smoking were available between 1991 and 2017. Education was classified into four categories: low (fewer than 9 years of compulsory school), medium (completed compulsory school), high (graduated from professional training or served an apprenticeship) and very high (qualified for university entrance), then collapsed to two groups: low to medium, and high to very high.

In total, there are 121 districts in Austria. Among the six stations in Austria which facilitate conscription medical examination covering nine provinces, Vienna is the most urban area with 3738 persons per km^2^ and a total of 1.8 million inhabitants, while population densities in other provinces (Burgenland, Carinthia, Lower Austria, Upper Austria, Salzburg, Styria, Tyrol, and Vorarlberg) are less than 2500 persons per km^2^ [23].

## Statistical analyses

We grouped years of conscription by 5‑year intervals in 1 wave (7 waves over 35 years) to provide stable figures for BMI subtypes with small counts. Descriptive statistics were analyzed in the most recent wave of conscript cohort (2013–2017) to present the contemporary status of education level, smoking behavior, and the 5th, 50th and 95th percentiles of BMI in each stratum of included factors.

To explore time trends in weight status from conscript cohort 1983–1987 to 2013–2017, we calculated the crude prevalence of class I–III and class III obesity in each conscript cohort by regions. Further, crude and multivariable adjusted logistic regression were used to evaluate time trend in classes I–III and class III obesity using conscript cohort as a predictor.

Next, we calculated the crude prevalence of obesity classes I–III and obesity class III in each conscript cohort (1991–1992 as reference group, 1993–1997 to 2013–2017) by subgroups defined by education and smoking (data on smoking are available from 1991 onwards). To explore the effect of education and smoking on the prevalence of obesity, multivariable adjusted logistic regressions were calculated including conscript cohort, age, smoking and education as independent and prevalence as dependent variable.

We tested for interaction effects between conscript cohort and education as well as smoking, respectively. Significant interactions were found for both education and smoking; therefore, the multivariable logistic regression was further grouped by level of education and smoking. Statistical significance for testing interaction and trends of Odds Ratio (OR) was set at *P* < 0.05. *P*‑values were not adjusted for multiple tests and should be interpreted explanatorily only. All analyses were performed using SAS version 9.4 (SAS Institute Inc., Cary, NC, USA).

## Results

Data on 1,507,040 young men were used for analyses. Over 1.5 million young men were grouped by 5‑year interval in 7 conscript cohorts, with the minimum size of 192,669 young men in each cohort. Among conscript cohort 2013–2017, obesity was prevalent (8.2%, 95% CI 8.1–8.3%) (Table 1). Majority of the conscript cohort had normal weight (66.2%, 95% CI 66.0–66.4%), did not smoke (56.4%) and had low to medium education (72.4%). The overall prevalence of obesity increased from 1.6% (95% CI: 1.6–1.7%) to 8.2% (95% CI: 8.1–8.3%) over 35 years. A more than 10-fold increase in prevalence from 0.04% to 0.5% was observed in obesity class III (Table 1).

**Table 1:** Characteristics of Austrian conscript cohorts between 1983 and 2017 (aged 17–19 years)

		Underweight (BMI < 18.5)		Overall obese I–III (BMI ≥ 30)		Obesity class III (BMI ≥ 40)	
	*N*	Prevalence %	95% CI	Prevalence %	95% CI	Prevalence %	95% CI
*Total*	1503740	5.98	5.94–6.02	4.80	4.76–4.83	0.24	0.23–0.25
*Age (years)*							
17	764889	6.60	6.55–6.66	4.48	4.43–4.52	0.22	0.21–0.23
18	738851	5.32	5.28–5.38	5.13	5.08–5.18	0.27	0.25–0.28
*Education*							
Low to medium	1089107	5.79	5.75–5.84	5.50	5.45–5.54	0.29	0.28–0.30
High to very high	410755	6.42	6.35–6.50	2.96	2.91–3.01	0.12	0.11–0.13
*Smoking*							
No	623344	6.32	6.26–6.38	4.86	4.80–4.91	0.26	0.25–0.28
Yes	482618	5.78	5.72–5.85	7.07	6.99–7.14	0.38	0.36–0.40
*Cohort*							
1983–1987	258294	5.81	5.72–5.90	1.63	1.58–1.68	0.04	0.03–0.05
1988–1992	221681	5.50	5.40–5.59	2.65	2.59–2.72	0.07	0.06–0.08
1993–1997	192823	5.57	5.47–5.67	3.88	3.79–3.97	0.15	0.14–0.17
1998–2002	206855	6.37	6.26–6.47	4.36	4.27–4.45	0.21	0.19–0.23
2003–2007	212456	6.21	6.11–6.31	6.26	6.16–6.36	0.31	0.28–0.33
2008–2012	218962	5.71	5.62–5.81	7.52	7.40–7.63	0.46	0.43–0.48
2013–2017	192669	6.77	6.66–6.88	8.18	8.06–8.30	0.54	0.51–0.58

*BMI* Body Mass Index, *CI* Confidence Interval

**Fig. 1:**
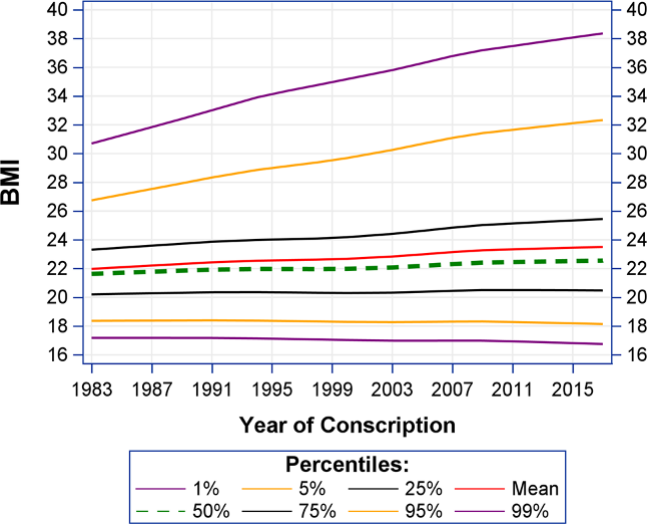
Time trends in BMI by percentiles among Austrian conscript between 1983 and 2017

In support, Fig. 1 illustrates that the most significant increases were observed in the higher BMI percentiles among Austrian conscripts from 1983 to 2017.

**Fig. 2:**
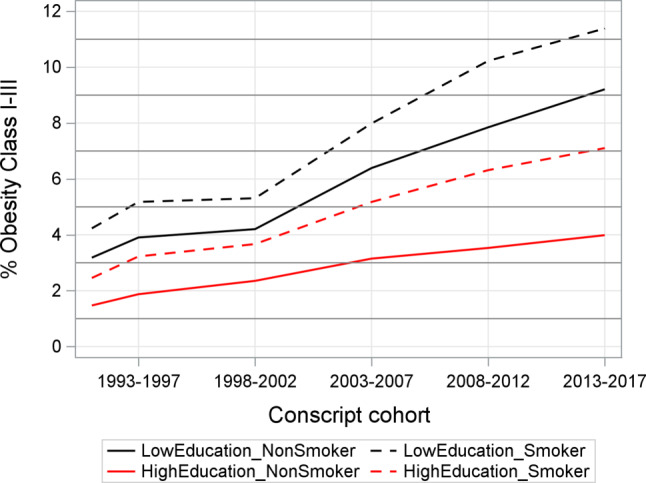
Trends in obesity class I–III by education and smoking status

In the logistic regression adjusting for year of birth and age only, class I–III obesity exhibited a significant increase from 1983–1987 to 2013–2017 (*p*-value for trends < 0.001). We observed consistent association between education level and smoking status with obesity prevalence across conscript cohort similar to Fig. 2. Owing to the significant interaction between conscript cohort, education and smoking, we presented the estimated ORs and 95% CI of conscript cohort by education and smoking status, adjusting for age from 1991 to 2017 (Table 2 and Fig. 2). Across all stations, we observed increased prevalence of obesity in both education groups (all *p*-values for trend < 0.001).

**Table 2:** Multivariable adjusted model for class I–III obesity by education and smoking status

	Low to median education		High to very high education	
	Non-smokers	Smokers	Non-smokers	Smokers
	OR (95% CI)	OR (95% CI)	OR (95% CI)	OR (95% CI)
*Age (years)*				
17	1	1	1	1
18	1.20 (1.16 to 1.24)	1.14 (1.10 to 1.17)	1.45 (1.37 to 1.54)	1.39 (1.28 to 1.51)
*Cohort*				
1991–1992	1	1	1	1
1993–1997	1.23 (1.14–1.32)	1.25 (1.16–1.34)	1.28 (1.12–1.46)	1.36 (1.12–1.66)
1998–2002	1.34 (1.25–1.43)	1.28 (1.19–1.38)	1.60 (1.39–1.83)	1.55 (1.27–1.89)
2003–2007	2.09 (1.95–2.23)	1.99 (1.86–2.13)	2.16 (1.90–2.45)	2.22 (1.83–2.69)
2008–2012	2.59 (2.44–2.77)	2.61 (2.44–2.79)	2.42 (2.14–2.74)	2.73 (2.25–3.31)
2013–2017	3.10 (2.91–3.31)	2.94 (2.74–3.15)	2.77 (2.45–3.13)	3.09 (2.55–3.76)

*OR* Odds Ratio, *CI* Confidence Interval

## Discussion

In a population-based sample of young men, the prevalence of obesity has been rapidly increasing over the last three and half decades in Austria, suggesting an urgent need of preventive measures to curb this trend. By 2017, only 64.9% young men in Austria were with normal weight. The prevalence of overweight and obesity increased in the overall Austrian young men, and in each subgroup defined by education and smoking status. Nevertheless, the prevalence of obesity is constantly higher among those with low vs. high education level, and smokers vs. non-smokers. Importantly, the difference in prevalence of obesity between education levels and smoking status appeared to widen over time.

Excessive weight and the simultaneous increase in obesity-related chronic conditions lead to premature mortality, morbidity, and long-term disability [29, 30]. Hence, a significant proportion of healthcare, economical, and societal burdens associated with chronic diseases could be potentially reduced by managing weight status. The 2015 World Health Organization Commission reported that adolescence can be a critical time or excess weight gain [31].

In the present study, we report rapidly increasing obesity in young men aged 17–< 19 years in Austria over the past 35 years. A previous study using five Austrian nationally representative cross-sectional survey data reported an increasing trend in adults BMI and prevalence of obesity (BMI > 30 kg/m^2^) from 1973 to 2007, particularly among the low educational groups, [17] and men in western Austria [18]. Another survey study was conducted among Austrian farmers in 1999/2000, reporting the prevalence of obesity peaked at 15–19 years old, and the eastern areas of Austria [16]. Although both studies were limited by using self-reported measures, another two Austria-based studies reported routinely collected national data on objectively measured weight and height among male conscripts. Based on the most recent study, the relevance of overweight and obesity among Austrian young men were 20.57% and 8.36%, respectively, in 2010 [15]. Both conscript data-based studies found an increasing gradient of higher BMI from west to east of Austria [14]. Although these data are striking, none of the previous studies considered smoking and its interaction with education.

We observed socioeconomic inequalities, similar to those reported in other developed countries. For instance, lower level of education or urbanization appeared to be associated with larger body size in US children and adolescents [32] and adults [33]. Whereas for the UK children and adolescents, the observed BMI socioeconomic inequalities were historically small but widened overtime [34]. Notably, our data suggested a widening gap in obesity prevalence across sociodemographic groups defined by education level and smoking status.

In this study only smoking and education have been included which may be seen as a potential limitation. Both variables are good proxies for sociodemographics thereby adjusting for other effects like alcohol consumption and physical activity to some extent. Furthermore, data on alcohol consumption and physical activity were not collected and therefore we were not able to include those variables. Still, we think that smoking and education status are able to provide an unbiased estimate for the prevalence of obesity.

In conclusion, the prevalence of obesity has been rapidly increasing in Austrian young men. Higher level of education appeared to be associated with lower prevalence of obesity, particularly among the non-smokers. Nevertheless, medical practice and public health policy should join forces in promoting the awareness of obesity in youth, and design population-level obesity prevention strategies and target interventions for the high-risk groups to reduce weight gaps within the population.
